# Unraveling the Glycosylation Machinery of *Bothrops jararaca* Snake Through Multiomics and Structural In Silico Analyses

**DOI:** 10.3390/ijms27188238

**Published:** 2026-09-16

**Authors:** Thales A. M. Fernandes, Milton Y. Nishiyama-Jr.

**Affiliations:** Laboratory of Applied Toxinology, Butantan Institute, São Paulo 05503-900, SP, Brazil; t.fernandes.proppg@proppg.butantan.gov.br

**Keywords:** glycans, glycosyltransferases, oligosaccharyltransferase, snake venom

## Abstract

Glycosylation is a ubiquitous post-translational modification (PTM) that influences folding, stability, and function of proteins. In eukaryotes, *N*-glycosylation corresponds to the linkage to asparagine (Asn), while *O*-glycosylation involves the linkage to serine (Ser) or threonine (Thr). Although glycosylation is one of the main PTMs of viperid snake venoms, the enzymatic machinery associated with toxin glycosylation remains poorly characterized in venomous organisms. To fill in this gap, we performed multiomics and structural in silico analyses of publicly available experimental genomic, transcriptomic, and proteomic datasets, combined with structural modeling, to investigate the glycosylation pathway of the medically important snake *B. jararaca*. The multiomics analysis suggested that the glycosylation machinery is coupled to the venom production cycle and influenced by sex-specific differences, underscoring the glycosylation heterogeneity between female and male specimens. In addition, comparative transcriptomics analysis highlighted different expression patterns of glycosyltransferases and glycosidases in different tissues. Moreover, structural analysis of the predicted oligosaccharyltransferases complexes and glycosylated toxins provided insights into the glycosylation pathway in the venom gland. Collectively, our study advances the understanding of the synthesis and processing of toxins and sheds light into the conservation and variability of the glycosylation machinery.

## 1. Introduction

Glycosylation is one of the most abundant and structurally diverse post-translational modifications (PTMs) [1,2]. Glycans play crucial roles in various physiological functions, and modifications in the glycome are linked to numerous diseases [3,4]. Moreover, the covalent attachment of oligosaccharides to Asn (*N*-glycosylation) or Ser/Thr residues (*O*-glycosylation) influences the protein folding stability, and activity [1,2].

In eukaryotes, *N*-glycosylation involves the assembly of a lipid carrier and the *en bloc* transfer of glycans to Asn residues by the oligosaccharyltransferase (OST) complex in the endoplasmic reticulum (ER), followed by the processing of *N*-glycans mediated by glycosyltransferases (GTs) and glycosidases (GHs) in the ER and Golgi complex [1,2]. In parallel, *O*-glycosylation occurs in the Golgi complex, where GTs add GalNAc residues from activated nucleotides (UDP-GalNAc) to Ser/Thr residues [5]. Subsequently, the glycans are processed by GTs and GHs, generating a wide diversity of glycans [1,2,5]. *N*-glycosylation plays important roles in the structure and activity of snake venom toxins, while the *O*-glycosylation remains poorly studied [6,7,8,9,10,11,12]. In this context, since glycosylation is important in various biological phenomena, a detailed characterization of the glycosylation machinery offers a deeper understanding of the synthesis and processing of snake venom toxins.

The molecular basis and the evolving view of the asparagine-linked glycosylation have been investigated through structural analyses of the central component of the *N*-glycosylation pathway and multimeric membrane-bound OST complexes [13]. Nonetheless, determining the tertiary structure of membranous proteins is often time-consuming, low-throughput, and difficult for complex structures [14,15]. In this context, deep learning methods have revolutionized the field by accurately predicting three-dimensional structures from the amino acid sequences, enabling the investigation of complex structures difficult to solve experimentally, and improving the comprehension of the structural basis of key biological phenomena [16,17,18].

In this work, we investigated the putative glycosylation machinery in *B. jararaca* by integrating public experimental multiomics and structural in silico analyses. Rather than aiming to biochemically validate individual enzymes, our study maps and prioritizes candidate glycosyltransferases, glycosidases, and OST-associated components potentially involved in venom gland protein processing. We also explored venom gland activation, sex-associated variation, tissue-associated expression patterns, and predicted glycosylation features of snake venom toxins. Collectively, our study contributes to a better understanding of the synthesis and processing of snake venom toxins, and sheds light into the conservation and variability of the glycosylation machinery.

## 2. Results and Discussion

### 2.1. B. jararaca Venom Gland Glycosylation Machinery

**Figure 1:**
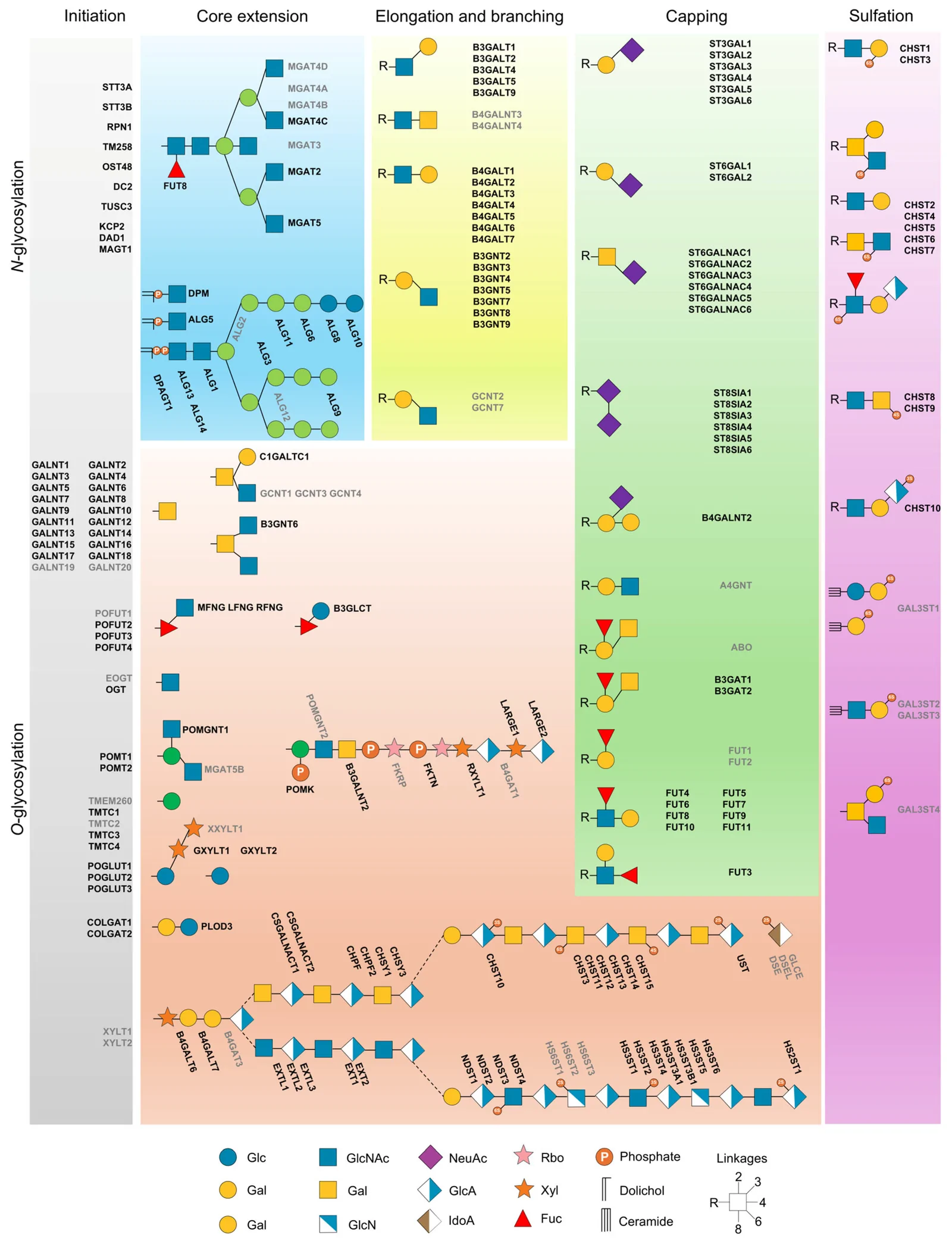
Schematic view of the glycosylation pathway in *B. jararaca* venom gland. The *N*-glycosylation starts in the ER, involving the LLO-synthesis and the en bloc transfer by the oligosaccharyltransferase (OST) complexes to Asn residues. In parallel, *O*-glycosylation occurs in the Golgi complex, where glycosyltransferases add GalNAc residues to Ser/Thr residues. After the protein is glycosylated, the glycans are processed by glycosidases, glycosyltransferases, and other enzymes responsible for core elongation, branching, capping, and modification of the glycans in the secretory pathway. The common enzymes shared between *B. jararaca* and humans are colored in black, while the absent proteins in *B. jararaca* are colored in grey. The glycosylation pathway was adapted from Glycopacity (https://glyco.me/glycopacity, accessed on 29 Janurary 2025).

The diversity of glycan structures is ultimately determined by the composition of GTs and GHs [19]. In *B. jararaca* venom gland, we identified a broad repertoire of approximately 180 families of putative enzymes associated with synthesis and processing of glycans (Supplementary File S2). The *N*-glycan structures of *B. jararaca* consist of high-mannose and hybrid/complex oligosaccharides, possessing terminal residues of sialic acid (Neu5Ac and Neu5Gc), *N*-acetylglucosamine (GlcNAc), mannose (Man), core residues of galactose (Gal), and fucose residues (Fuc) [11]. Corroborating with these observations, we identified sialyltransferases, *N*-acetylglucosaminyltransferases, and fucosyltransferases in the glycosylation machinery of *B. jararaca* venom gland (Figure 1; Supplementary File S2). Importantly, the terminal sialic acid residues in snake venom toxins are pointed out to mediate a broad range of biological phenomena, including proteolysis and interaction with cellular targets [6,7,8,9,10,11,12]. In addition, we identified putative sulfotransferases and xylosyltransferases responsible, respectively, for the modification of glycans and transfer of xylose (Xyl) residues, which has been observed in trocarin, a blood coagulation factor Xa homologue from venom of the Australian elapid *Tropidechis carinatus* [20].

**Figure 2:**
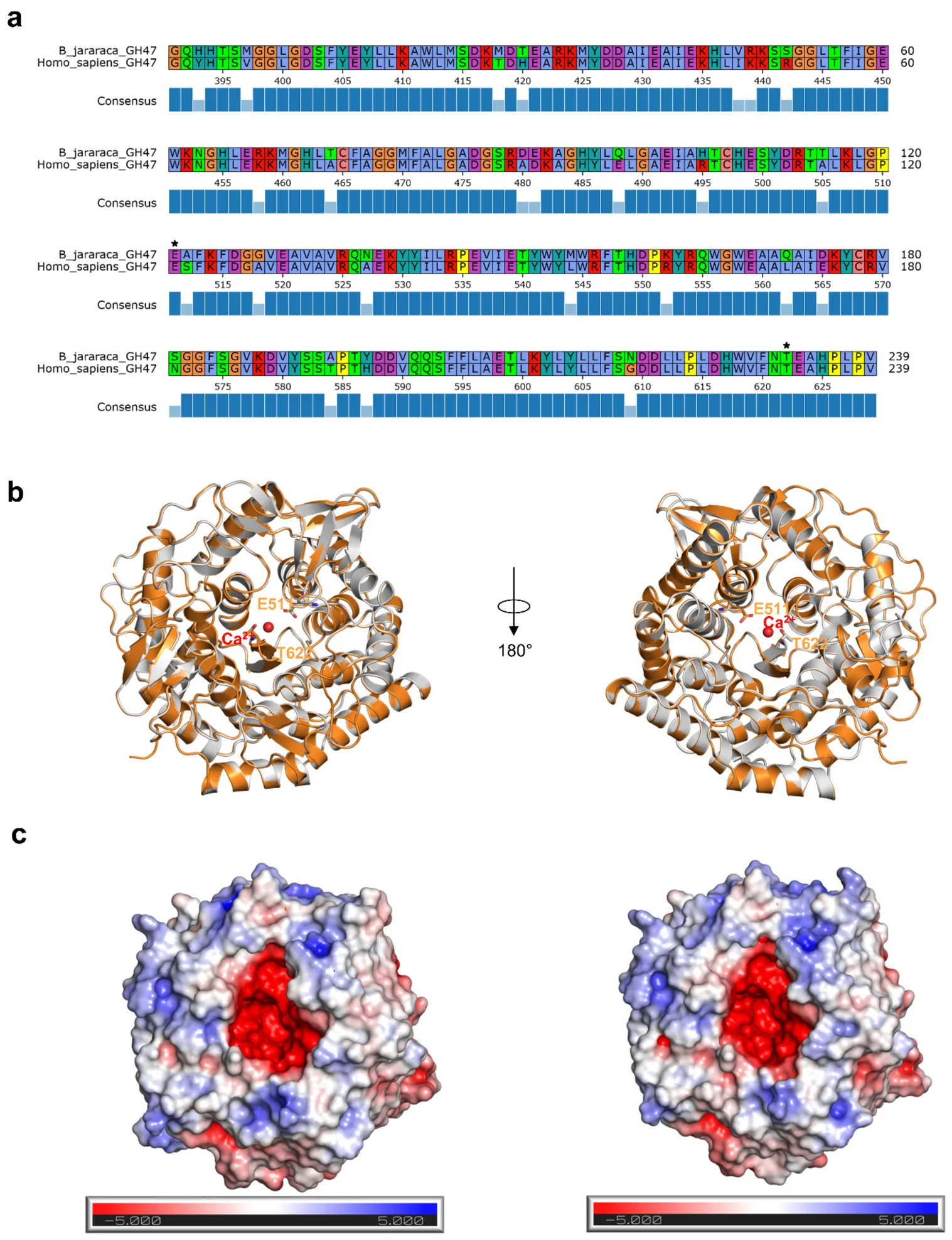
Structural analysis of *B. jararaca* alpha-1,2-mannosidase (GH47). (**a**) Sequence alignment between *B. jararaca* and human enzymes. The catalytic residues are highlighted by asterisks. (**b**) Structural superposition between *B. jararaca* (orange) and human (grey) enzymes. The catalytic residues are shown in sticks, with carbon, oxygen, and nitrogen atoms colored grey, red, and blue, respectively. (**c**) Electrostatic surface of *B. jararaca* (left) and human (right) enzymes.

Furthermore, by aligning the protein-coding sequences of *B. jararaca* within the venom gland and the human glycosylation machinery, we found a high conservation of enzymes associated with the initiation, core extension, elongation, branching, and capping (Figure 1; Supplementary File S3). The differences in the glycosylation machinery could be associated with the presence of specializations in the venom gland. Although we did not conduct experimental analysis for the annotation of the putative enzymes, we identified the presence of biochemically characterized domains in the putative *B. jararaca* glycosidases (Figure 2; Supplementary File S4). For example, by aligning the sequences of the putative mannosyl-oligosaccharide alpha-1,2-mannosidase (GH47) from *B. jararaca* and the experimentally characterized enzyme, we verified that the sequences possess an identity of 88.12% (Figure 2; Supplementary File S4). Moreover, we observed in the structural superposition the conservation of the catalytic residues and the electrostatic surface in the active site (Figure 2b,c), supporting prioritization of these proteins as candidate glycosylation-related components. In this sense, the annotation of the putative GTs and GHs underscored the glycosylation machinery of *B. jararaca*, which could be further extended by recombinant expression and experimental assays to evaluate the enzymatic activity and substrate specificity of the putative enzymes.

### 2.2. Quantitative Transcriptomic Analysis

**Figure 3:**
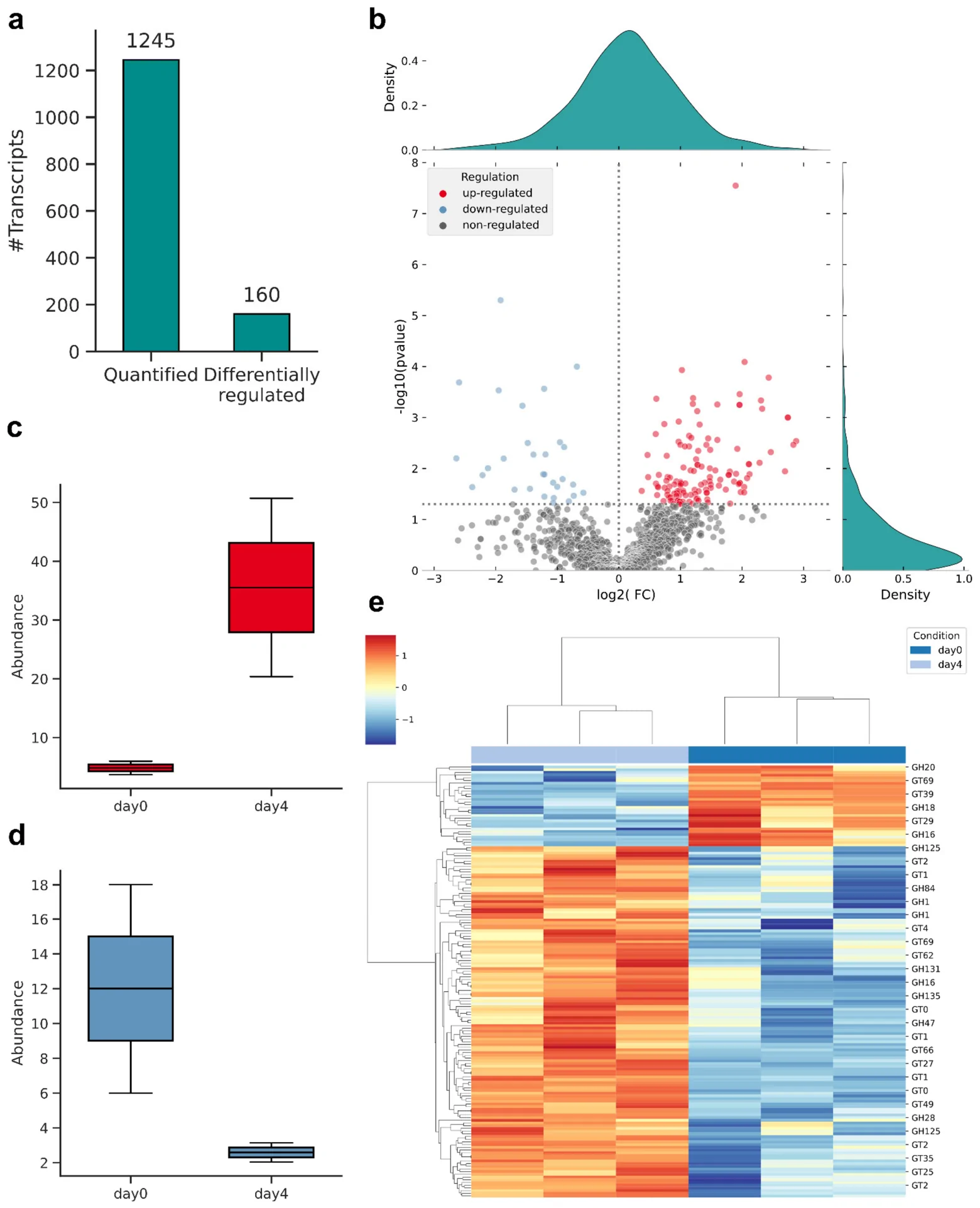
Quantitative transcriptomic analysis. (**a**) Bar plot displaying the total number and differentially regulated transcripts. (**b**) Volcano plot highlighting the differentially regulated transcripts in the venom glands of snakes that had no venom previously removed (0-day group) and venom glands collected four days after venom extraction (4-day group). (**c**,**d**) Boxplot depicting up- and down-regulated GT49 (glycosyltransferase) and GH27 (galactosidase) involved in the elongation and trimming of glycans, respectively. (**e**) Heatmap of differentially regulated transcripts between the 0-day and 4-day groups. For each experiment, the biological replicates (*n* = 3) were analyzed using independent *t*-test with Benjamini–Hochberg correction and statistical differences were considered for *p* < 0.05.

The dynamic rearrangement in the proteome landscape of *B. jararaca* venom gland has shown the existence of differentially abundant proteins involved in the venom production [21,22]. Interestingly, the abundance of toxins in the venom gland of *B. jararaca* shifts during the venom production, and proteins from the ER and cytoplasm are more abundant during the synthesis and secretion of toxins [21,22]. In our study, we observed a differential expression of GTs and GHs between venom glands of snakes that had no venom previously removed (0-day group) and venom glands collected four days after venom extraction (4-day group) (Figure 3). The quantitative transcriptomic analysis resulted in the identification of 1245 transcripts and 160 differentially regulated transcripts (Figure 3a). In addition, we verified that 130 transcripts were up-regulated and 30 down-regulated (Figure 3b), such as the GT49 and GH27 involved in the elongation and trimming of glycans, respectively (Figure 3c,d). Hierarchical clustering analysis of the differentially expressed transcripts highlighted differences between the 0-day and 4-day groups, suggesting transcriptional remodeling of glycosylation-related candidates during venom gland activation. However, we acknowledge that a larger number of biological replicates would improve statistical power, increase the robustness of the differential expression analysis, and enhance the reliability of the identified differentially abundant transcripts. Nevertheless, our findings are consistent with the proteomic analyses, and the observed differential expression patterns likely reflect biologically relevant changes associated with venom production and the dynamic regulation of the glycosylation machinery in *B. jararaca*.

### 2.3. Quantitative Proteomic Analysis

**Figure 4:**
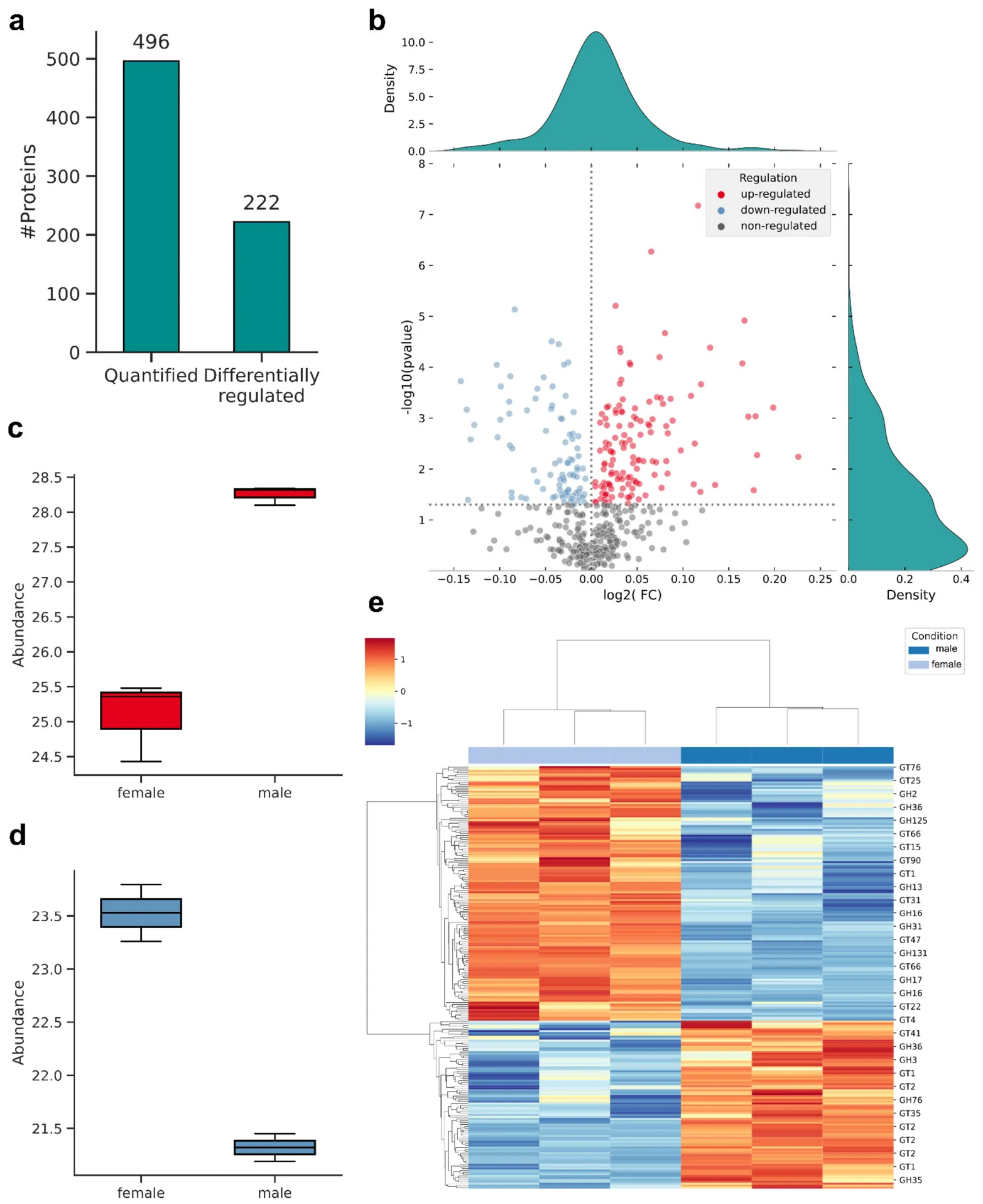
Quantitative proteomic analysis. (**a**) Bar plot displaying the total number and differentially abundant proteins. (**b**) Volcano plot highlighting the differentially regulated proteins by comparing the abundance of proteins in the female and male specimens. (**c**,**d**) Boxplot depicting up- and down-regulated GT2 (glycosyltransferase) and GH5 (mannosidase) involved in the elongation and trimming, respectively. (**e**) Heatmap of differentially regulated proteins between the venom glands of female and male specimens. For each experiment, the biological replicates (n = 3) were analyzed using independent *t*-test with Benjamini–Hochberg correction and statistical differences were considered for *p* < 0.05.

In *B. jararaca*, despite the similarity in the protein level, the venoms of female and male specimens differ in the protein glycosylation [23]. In accordance, we verified that GTs and GHs were differentially abundant between the venom glands of female and male specimens (Figure 4). From 496 quantified GTs and GHs, 134 proteins were up-regulated and 88 proteins down-regulated (Figure 4a,b). For example, GT2 and GH5 involved in the glycan elongation and trimming were up- and down-regulated, respectively (Figure 4c,d). In addition, clustering analysis evidenced the separation between the female and male specimens according to the differentially abundant GTs and GHs (Figure 4e). Since glycosylation can influence the protein structure and function, the observed sex-associated differences in the venom gland glycosylation machinery may have ecological implications, such as in prey–predator interactions. Interestingly, the protein glycosylation is involved in the interaction of post-synaptic nicotinic acetylcholine receptors (nAChR) of Australian agamid lizards and snake venom α-neurotoxins [24]. Moreover, it was observed that venoms from females are more potent for hemorrhagic activities, whereas venoms from males are more potent for coagulant activities in *B. jararaca* [25]. In this context, our results highlight the glycosylation as an additional feature for the venom heterogeneity beyond differences in the expression and abundance of snake venom toxins. We acknowledge that the limited number of biological replicates reduces the statistical power of the differential abundance analysis. Future studies with larger sample sizes will improve statistical robustness, reduce the likelihood of false positives and false negatives, and enhance the confidence in the identified differentially abundant GTs and GHs. Moreover, cross-study comparisons to correlate the transcript and protein abundances across multiomic datasets should carefully address possible batch effects. Collectively, our findings suggest that the glycosylation machinery could be influenced by sex-specific differences, which may contribute to the venom glycosylation heterogeneity, due to differences in the abundance of GTs and GHs involved in the synthesis and processing of glycans.

### 2.4. Comparative Transcriptomic Analysis

**Figure 5:**
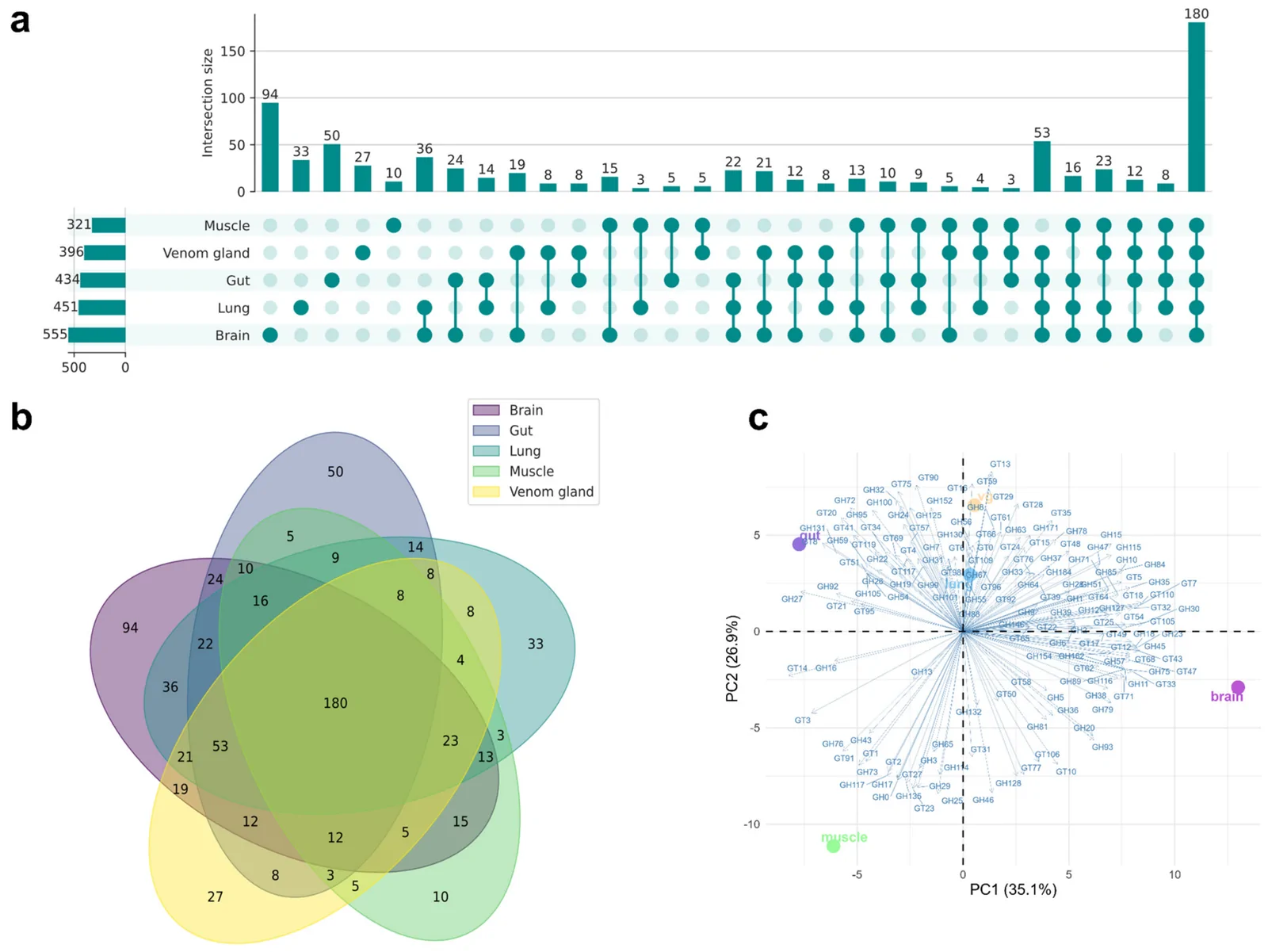
Comparative transcriptomic analysis. (**a**) Upset plot showing the overlap of glycosyltransferases (GTs) and glycosidases (GHs) among the five analyzed tissues (brain, gut, lung, muscle, and venom gland) of *B. jararaca*. The bars correspond to the number of GTs and GHs. (**b**) Venn diagram depicting the common and unique enzymes across the five tissues. (**c**) Principal Component Analysis (PCA) illustrating the clustering of tissues based on the GTs and GHs.

The expression of GTs and GHs is known to be distinct across cells and tissues [26]. In venomous snakes, the global view of tissue transcript expression is poorly described [27]. In our study, by comparing the composition of carbohydrate-active enzymes (CAZymes) in the brain, gut, lung, muscle, and venom gland of *B. jararaca*, we detected specific differences in the putative GTs and GHs (Figure 5a,b). Furthermore, clustering analysis underscored distinct patterns among the tissues (Figure 5c). Collectively, the differences in the body tissue expression could be associated with the specific roles of the glycans. Nevertheless, since the transcriptomic datasets derived from bulk tissues, single-cell and temporal analyses could further extend the understanding of the diversity of the GTs and GHs repertoire across *B. jararaca* tissues.

### 2.5. Structural Analysis of the Protein Glycosylation in B. jararaca Venom Gland

**Figure 6:**
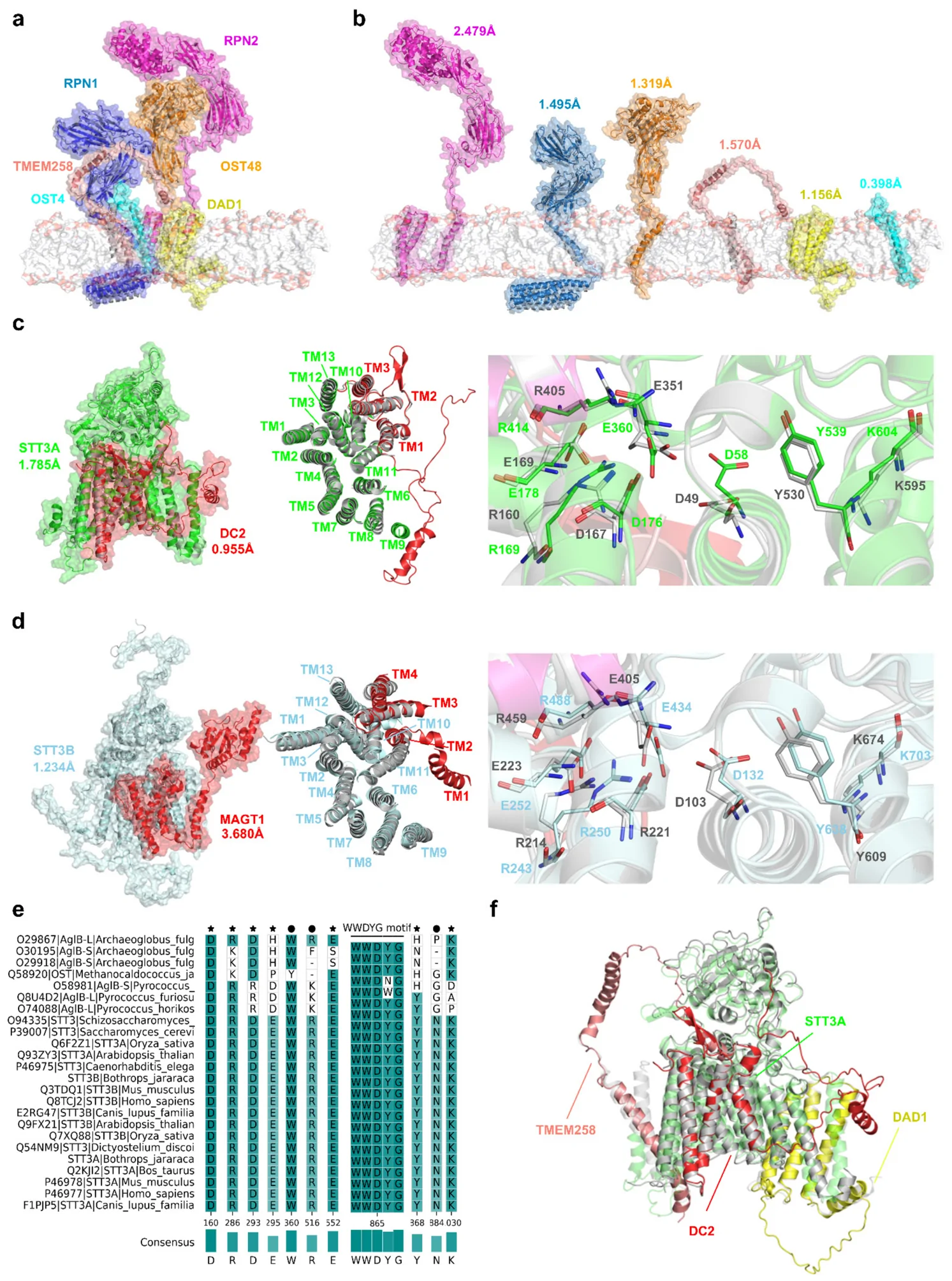
Structural analysis. (**a**,**b**) Superposition of *B. jararaca* OSTs non-catalytic subunits with the human counterparts. Proteins are represented in cartoons with the surfaces in the background. (**c**,**d**) Structural superposition of *B. jararaca* and human STT3A-DC2 and STT3B-MAGT1 catalytic subunits, respectively. In the center, the transmembrane domains are shown in cartoons. In the right, the catalytic and LLO-binding residues are shown as sticks, with carbon, oxygen, and nitrogen atoms colored grey, red, and blue, respectively. (**e**) Multiple Sequence Alignment (MSA) of the STT3 orthologs from *B. jararaca*. The WWDYG motif is highlighted as well as the conserved residues involved in catalysis and lipid-linked oligosaccharide (LLO) binding of STT3A (asterisks) and STT3B (circles). (**f**) Superposition of *B*. *jararaca* non-catalytic subunits DAD1, DC2, TMEM258, and the catalytic subunit STT3A with the human counterparts.

By deciphering the protein sequences and incorporating co-evolutionary information, recent advances in the field of computational structural biology have opened new avenues to investigate fundamental biological processes [28,29]. A pivotal step of the *N*-glycosylation pathway is the OST-catalyzed transfer of the oligosaccharide to the acceptor asparagine [30,31,32]. As the nascent polypeptides pass through the ER translocon, DC2 interacts with STT3A and mediates the co-translation glycosylation [33,34,35]. In parallel, MAGT1 and TUSC3 oxidoreductases interact with STT3B and mediate the post-translational glycosylation of sequons that have been skipped by the DC2-containing complex [36,37]. In addition, the non-catalytic subunits RPN1, RPN2, OST48, DAD1, OST4, and TMEM258 are required for the *N*-glycosylation, participating in the assembly, stability, and substrate selectivity of eukaryotic OST complexes [38,39,40,41,42]. In our work, we observed that *B. jararaca* non-catalytic subunits resemble the human counterparts (Figure 6a,b). Moreover, we observed that the transmembrane domains of *B. jararaca* STT3A and STT3B, as well as the residues involved in the catalytic cycle are highly conserved, such as the WWDYG motif (Figure 6c–e). In addition, by aligning the primary structure of STT3 orthologues, we identified a conservation of functional motifs, underscoring the preservation of catalytic residues (Figure 6e). Notably, we detected differences in the primary structures of the non-catalytic and catalytic subunits (Supplementary File S7; Supplementary Figures S1–S11). Particularly, *B. jararaca* DAD1, DC2, and TMEM258 subunits contain an extended N-terminal region that spans the catalytic site (Figure 6f). However, the relatively low iPTM scores predicted for TMEM258 indicate lower confidence in its protein–protein interfaces, and these structural models should therefore be interpreted with caution pending experimental validation. Despite the mechanistic impact of the differences in the catalytic and non-catalytic subunits requiring further analyses, the observed structural differences could affect the occupancy and selectivity of the *N*-glycosylation sequons, providing a means for the glycosylation of toxins in the venom gland. In addition, molecular dynamics (MD) simulations in a lipid bilayer environment could provide additional information on the conformational stability and dynamics of OST active sites and associated protein–protein interfaces.

**Figure 7:**
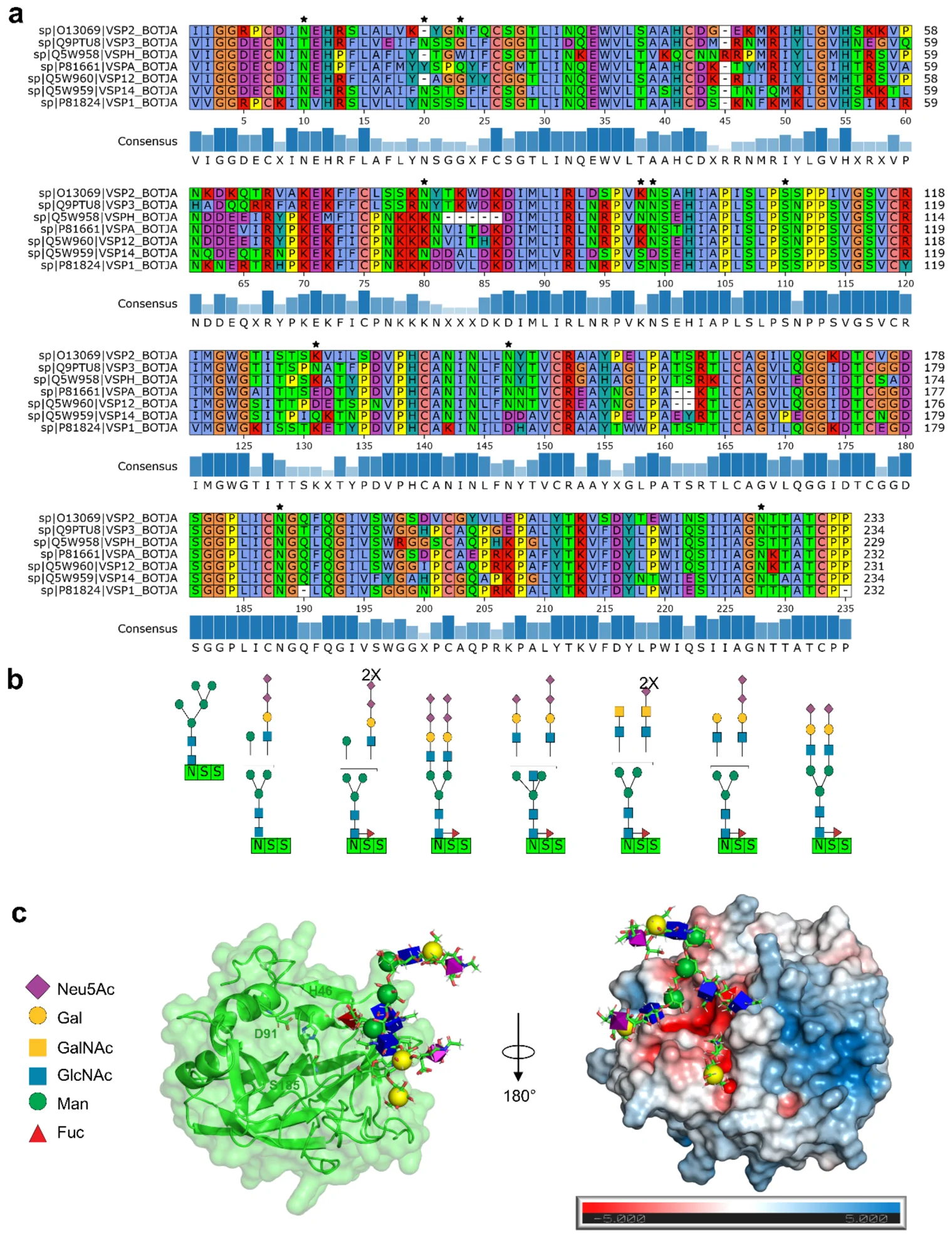
Structural analysis of *B. jararaca* SVSPs. (**a**) Multiple Sequence Alignment (MSA) between *B. jararaca* SVSPs. The putative glycosylation sites are highlighted by asterisks. (**b**) *N*-glycans present in the glycopeptides present in *B. jararaca* PA-BJ. (**c**) Structural prediction of PA-BJ. On the left, the catalytic residues are highlighted in sticks, with the carbon, oxygen, and nitrogen atoms colored in green, red, and blue, respectively. The electrostatic surface of PA-BJ is highlighted on the right.

In snake venom serine proteases (SVSPs), the glycosylation can influence the structural stability, solubility, substrate selectivity, as well as the ability to evade inhibitors [43]. Moreover, the carbohydrate moieties possess a high heterogeneity, varying in the monosaccharide composition, linkage, and branching [44]. In our work, by analyzing the primary structures of *B. jararaca* SVSPs, we identified high-conservation candidates of *N*- and *O*- glycosylation sites (Figure 7a). The similarity of the glycosylation sites could be associated with a convergent functional role. In addition, we noticed that the putative *N*-glycosylation sites are found in non-canonical N-X-S/T sequons (Figure 7a). The divergent glycosylation sites found in *B. jararaca* SVSPs could be associated with the structural differences found in the catalytic and non-catalytic subunits of *B. jararaca* OST, which are involved in the selectivity of the *N*-glycosylation sequons. Moreover, by analyzing the glycopeptides derived from PA-BJ, a thrombin-like enzyme from *B. jararaca*, we observed a microheterogeneity of *N*-glycans occupying the same putative glycosylation site (Figure 7b). The diversity of glycans structures could be related to the affinity of the toxins to recognize different targets [45]. In addition, the analysis of the tertiary structure of PA-BJ highlights the occupation of the *N*-glycans next to the active site (Figure 7c), which can influence the interaction with macromolecular targets [43]. Collectively, our findings highlight the importance of glycosylation as a conserved yet structurally diverse post-translational modification that contributes to the functional diversification of snake venom toxins.

## 3. Materials and Methods

### 3.1. Datasets

The transcriptomic and proteomic analyses were performed using publicly available experimental datasets. For the analysis of the expression of the glycosylation machinery during the venom production cycle, we used the venom gland transcriptome dataset (*n* = 3) obtained from 0-day and 4-days post-venom extraction [46]. This dataset is publicly available in the NCBI under the accession PRJNA262899 [46]. In parallel, for the comparative transcriptomic analysis of the glycosylation machinery across tissues (*n* = 3), we collected the transcriptomic datasets of the brain, gut, lung, muscle, and venom gland of *B. jararaca* [47]. This dataset is publicly available in the National Center for Biotechnology Information (NCBI) under the accession PRJNA691605 [47]. For the comparative proteomic analysis of the female and male venom glands (*n* = 3), we collected the proteomic dataset present in ProteomeXchange [48] under accession PXD040239 [49].

### 3.2. Transcriptome Assembly

The transcriptome assembly was performed by first trimming low-quality reads and adapter sequences with Trimmomatic v.0.39 [50] and filtering out PhiX contaminants with BBDuk v.38 (https://sourceforge.net/projects/bbmap/, (accessed on 15 August 2026)). The transcriptome assembly was performed using rnaSPAdes v.3.13.1 [51]. The assembly quality was assessed with BUSCO v.5.3 [52], using the single-copy orthologous genes of eukaryotes (“eukaryota_odb10”), and rnaQUAST v.2.0 [53]. The assembly summary statistics are available in the Supplementary File S1.

### 3.3. Transcriptome Annotation

The redundant transcripts, highly similar and fragmented transcripts, were clustered using CD-HIT v.4.8 [54]. The resulting non-redundant sequences were submitted to identification of complete protein-coding regions using TransDecoder v.5.5.0 (https://github.com/sghignone/TransDecoder, (accessed on 15 August 2026)). The annotation was performed using dbCAN3 v.5.2.1 [55] against the Carbohydrate-Active enZYmes (CAZy) database [56]. The transcriptome annotation is available in the Supplementary File S2. In addition, we aligned *B. jararaca* GHs sequences to the experimentally characterized dataset of GHs within the CAZY database [57] in order to predict the presence of catalytic domains.

### 3.4. Comparative Transcriptomic Analysis

In order to gain insights into the variability of the glycosylation machinery, we compared the repertoire of GTs and GHs from the transcriptome datasets of the brain, gut, lung, muscle, and venom gland of *B. jararaca* to visualize the intersections and tissue-specific distributions of CAZymes in the five tissues. In addition, we aligned the protein-coding regions of *B. jararaca* venom gland using diamond v.2.1.11 [58] with an e-value equal to 10^−5^ against the human glycosylation machinery from Glycopacity [59]. The sequence alignment is available in the Supplementary File S3.

### 3.5. Quantitative Transcriptomic and Proteomic Analyses

To investigate the dynamic rearrangement of the glycosylation machinery in the *B. jararaca* venom gland, we quantified the transcripts in the venom glands (*n* = 3) of snakes that had no venom previously removed (0-day group) and venom glands collected four days after venom extraction (4-day group) [46]. The paired-end reads were aligned to the venom gland transcriptome and the abundances estimated using RSEM v.1.3.1 [60]. In parallel, we quantified the level of proteins in the venom gland proteomes (*n* = 3) of female and male specimens of *B. jararaca* [49]. The raw spectra were analyzed with MSFragger v.4.0 in FragPipe v.23.0 using the LBQ-MBR workflow [61]. Afterwards, the quantitative transcriptomic and proteomic data were submitted for differential analysis using OmicScope v.1.4.6 [62]. For each experiment, the biological replicates (n = 3) were analyzed using independent *t*-test with Benjamini–Hochberg correction and statistical differences were considered for *p* < 0.05. The differentially quantified transcripts and proteins are available in the Supplementary Files S5 and S6, respectively.

### 3.6. Structural Analysis

To evaluate the structure of the central component of the *N*-glycosylation pathway, we first identified orthologs of the catalytic subunits of *B. jararaca* using blastp v.2.16 [63] with an e-value equal to 10^−5^ against UniProt-SwissProt [64]. A multiple sequence alignment (MSA) between the retrieved sequences was performed using MAFFT on the EMBL-EBI server [65]. The MSA was visualized using pyMSAviz v.0.4.0 (https://github.com/moshi4/pyMSAviz, (accessed on 15 August 2026)). The tertiary structures of *B. jararaca* OST complexes were modeled with AlphaFold v.3 [18], followed by structural relaxation using ROSIE v.2 [66], and quality assessment in the SWISS-MODEL server [67]. The structural assessment is available in the Supplementary File S7 (Supplementary Figures S12 and S13). The presence of signal peptides and their cleavage sites were predicted using SignalP v.6.0 [68] and Phobius v.1.01 [69]. To improve the model readability, an ER-like membrane (POPC:18, PLPC:18, POPE:6. PSPE:6, SAPE:25, SAPC:6, SAPI:3, SLPI:3, OLPS:4, PSM:5, POPA:1, CHOL:5) [70] was built using CHARMM-GUI Membrane Builder [71,72]. In addition, we compared the three-dimensional structures of *B. jararaca* OSTs subunits with the human complexes OST-A (PDB: 6S7O) and OST-B (PDB: 6S7T) [73] obtained from the Protein Data Bank (PDB) [74] using PyMOL v.3.1 (Schrödinger, Inc., NY, USA). Furthermore, in order to gain insights into the *N*-glycosylation of snake venom toxins, we performed an MSA analysis of SVSPs annotated in UniProt-SwissProt [64] from *B. jararaca*. Next, by extracting the glycopeptides sequences PA-BJ, we further analyzed the microheterogeneity present in the *N*-glycosylation site [44]. Then, we predicted the tertiary structure of PA-BJ using AlphaFold v.3 [18], followed by structural relaxation using ROSIE v.2. [66] and restored the glycosylation using GlycoShape (https://glycoshape.org/, accessed on 14 June 2026) [75].

## 4. Conclusions

In summary, by integrating multiomics and structural computational analyses, we investigated the glycosylation machinery of *B. jararaca*. Our results suggest that GT and GH candidates display variability among tissues, female and male specimens, and during the venom production, supporting the hypothesis that the glycosylation machinery may contribute to venom protein processing and toxin heterogeneity. Furthermore, structural analysis of the predicted OST subunits indicates conservation of key catalytic motifs, together with divergent regions that may influence the glycosylation of snake venom toxins in the venom gland. Rather than representing an endpoint, these findings establish a foundation for future experimental studies using recombinant expression, enzymatic assays, glycomics, and glycoproteomics to define catalytic activity, substrate specificity, glycan structures, and toxin site occupancy in the venom gland. In addition, since glycosylation can affect toxin structure and function, understanding the glycosylation pathway in the venom gland may be helpful for antivenom development or design of toxin-derived therapeutic molecules. Overall, our study advances the understanding of venom production and provides valuable insights for future investigations exploring the synthesis and processing of snake venom toxins.

## Data Availability

The transcriptomic data is available in NCBI under the BioProject accessions PRJNA691605 and PRJNA262899, and the proteomic data is available in ProteomeXchange under PXD040239.

## Supplementary Materials

The following supporting information can be downloaded at: https://www.mdpi.com/article/10.3390/ijms27188238/s1.
