# Supplementary material for: Unraveling the Glycosylation Machinery of Bothrops jararaca Snake Through Multiomics and Structural In Silico Analyses
Source: Int J Mol Sci. 2026 Sep 16;27(18):8238. doi: 10.3390/ijms27188238 (PMC13607862; doi:10.3390/ijms27188238)
Supplement: Supplementary file 1 [file ijms-27-08238-s001.zip › Supplementary_File S7.pdf]

Supplementary File S7

for

Unraveling the Glycosylation Machinery of *Bothrops jararaca* Snake Through Multiomics and Structural In Silico Analyses

Thales A. M. Fernandes and Milton Y. Nishiyama-Jr. *

Laboratory of Applied Toxinology, Butantan Institute, São Paulo 05503-900, SP, Brazil; t.fernandes.proppg@proppg.butantan.gov.br
* Correspondence: milton.nishiyama@butantan.gov.br

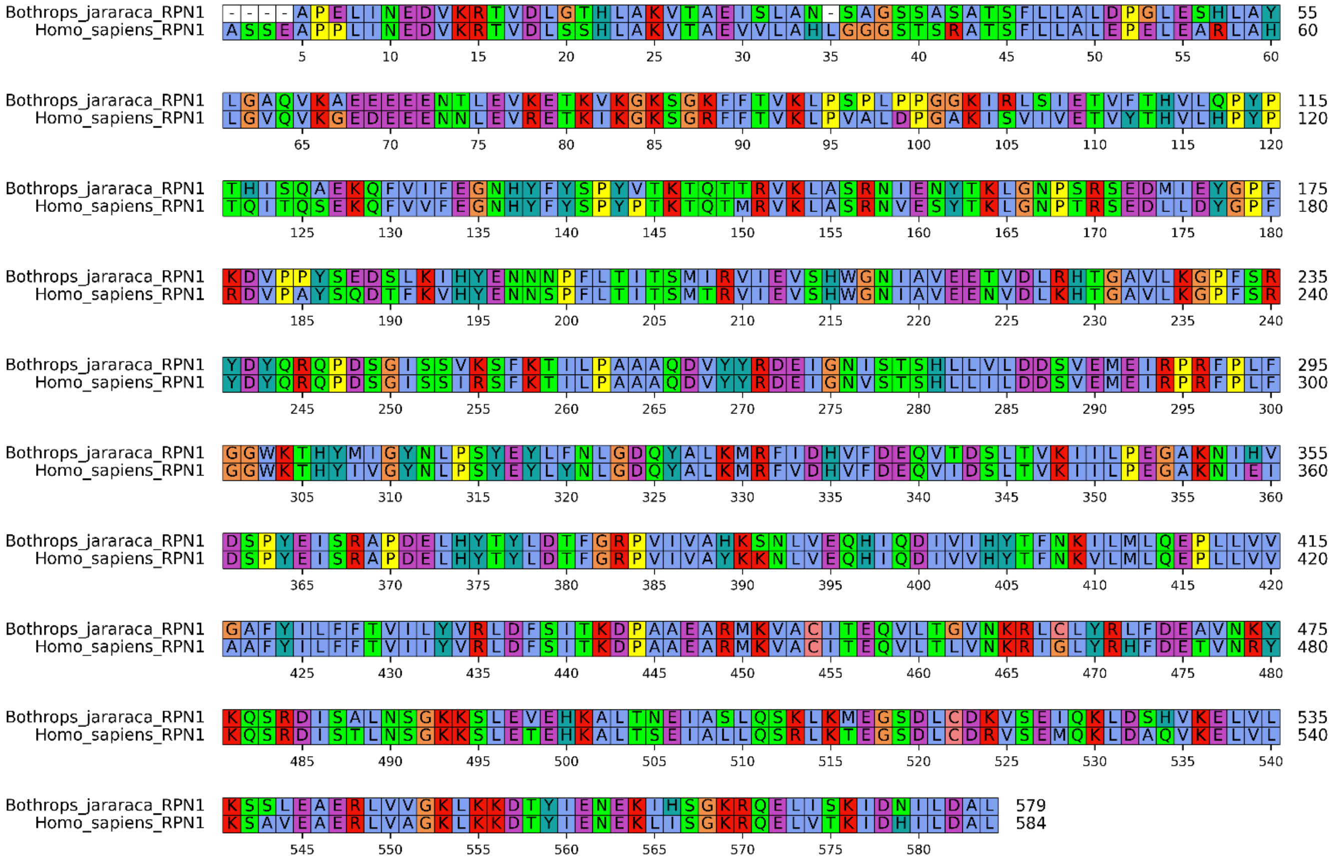

Figure S1. Sequence alignment between RPN1 from *B. jararaca* and humans (UniProt/Swiss-Prot: P04843).

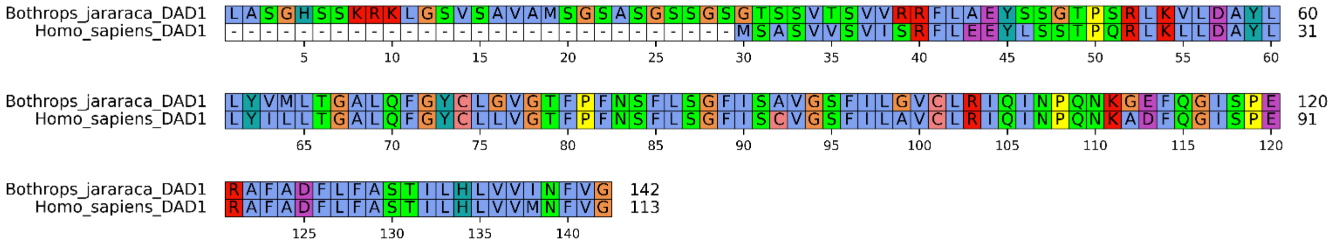

Figure S2. Sequence alignment between DAD1 from *B. jararaca* and humans (UniProt/Swiss-Prot: P61803).

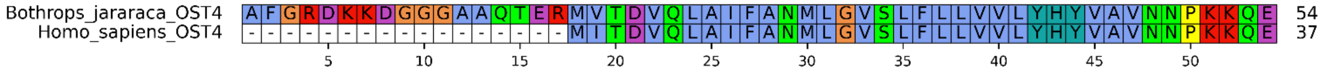

Figure S3. Sequence alignment between OST4 from *B. jararaca* and humans (UniProt/Swiss-Prot: P0C6T2).

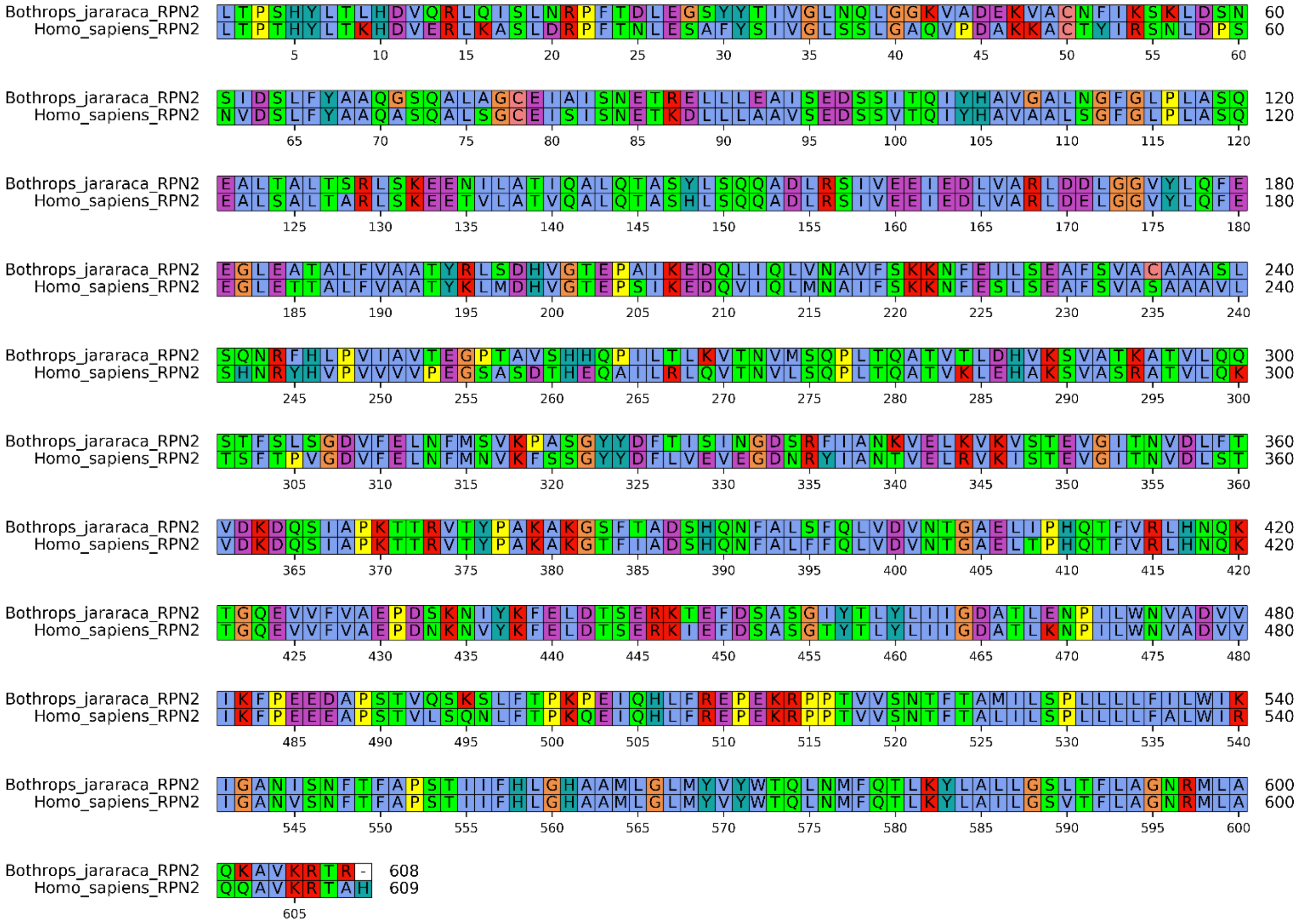

Figure S4. Sequence alignment between RPN2 from *B. jararaca* and humans (UniProt/Swiss-Prot: P04844).

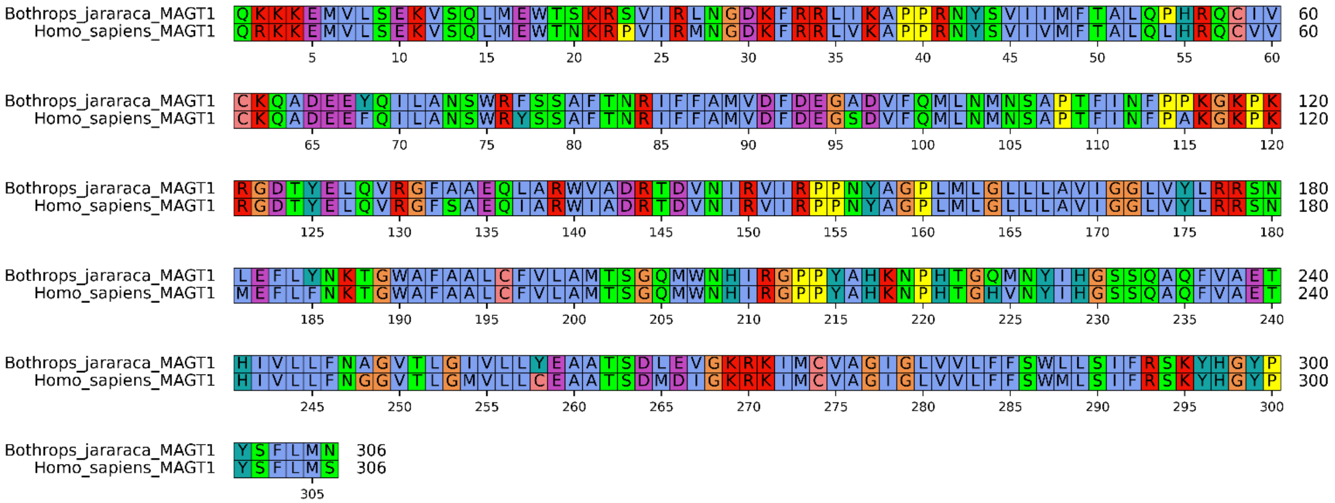

Figure S5. Sequence alignment between MAGT1 from *B. jararaca* and humans (UniProt/Swiss-Prot: Q9H0U3).

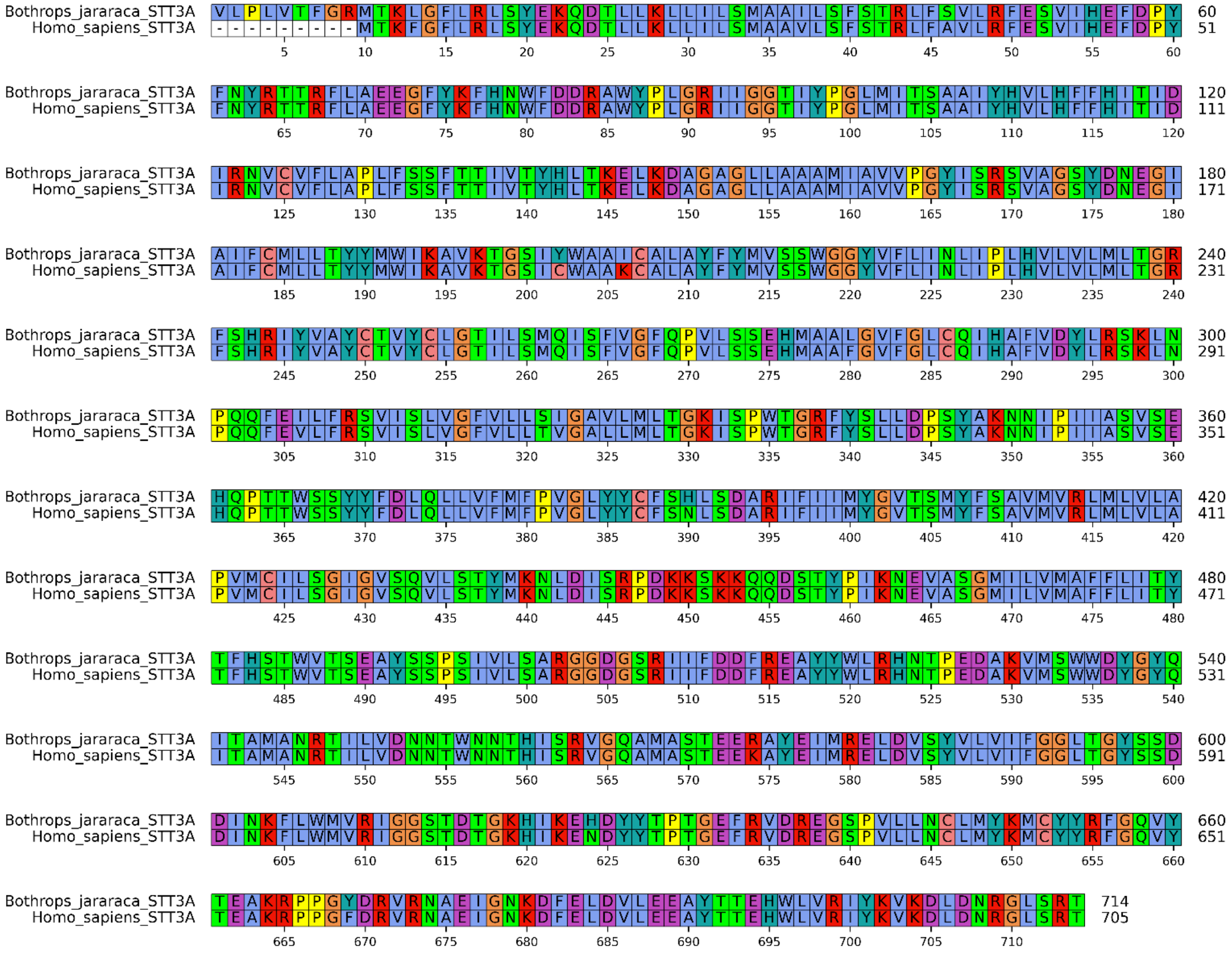

Figure S6. Sequence alignment between STT3A from *B. jararaca* and humans (UniProt/Swiss-Prot: P46977).

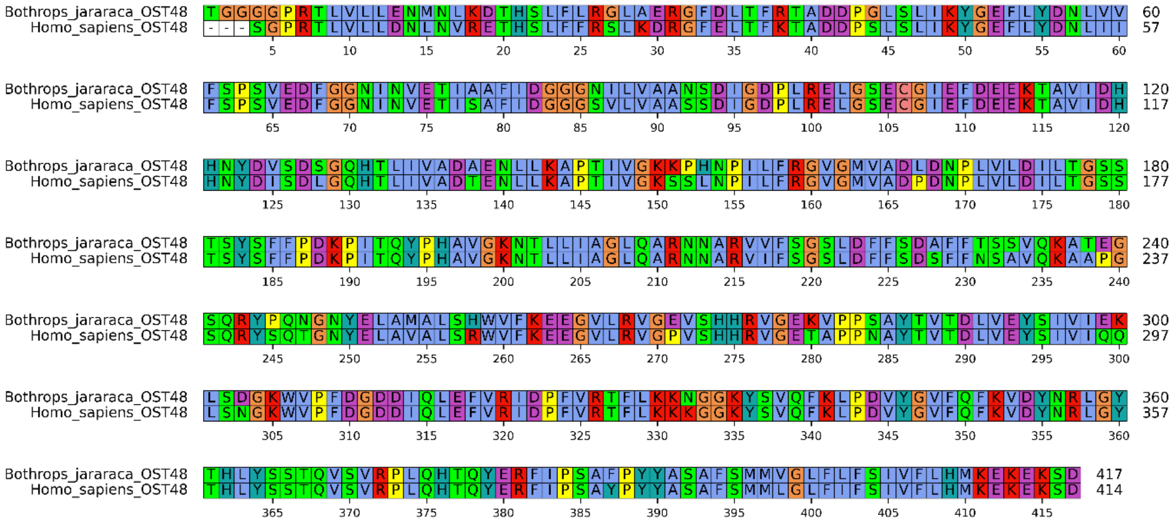

Figure S7. Sequence alignment between OST48 from *B. jararaca* and humans (UniProt/Swiss-Prot: P39656).

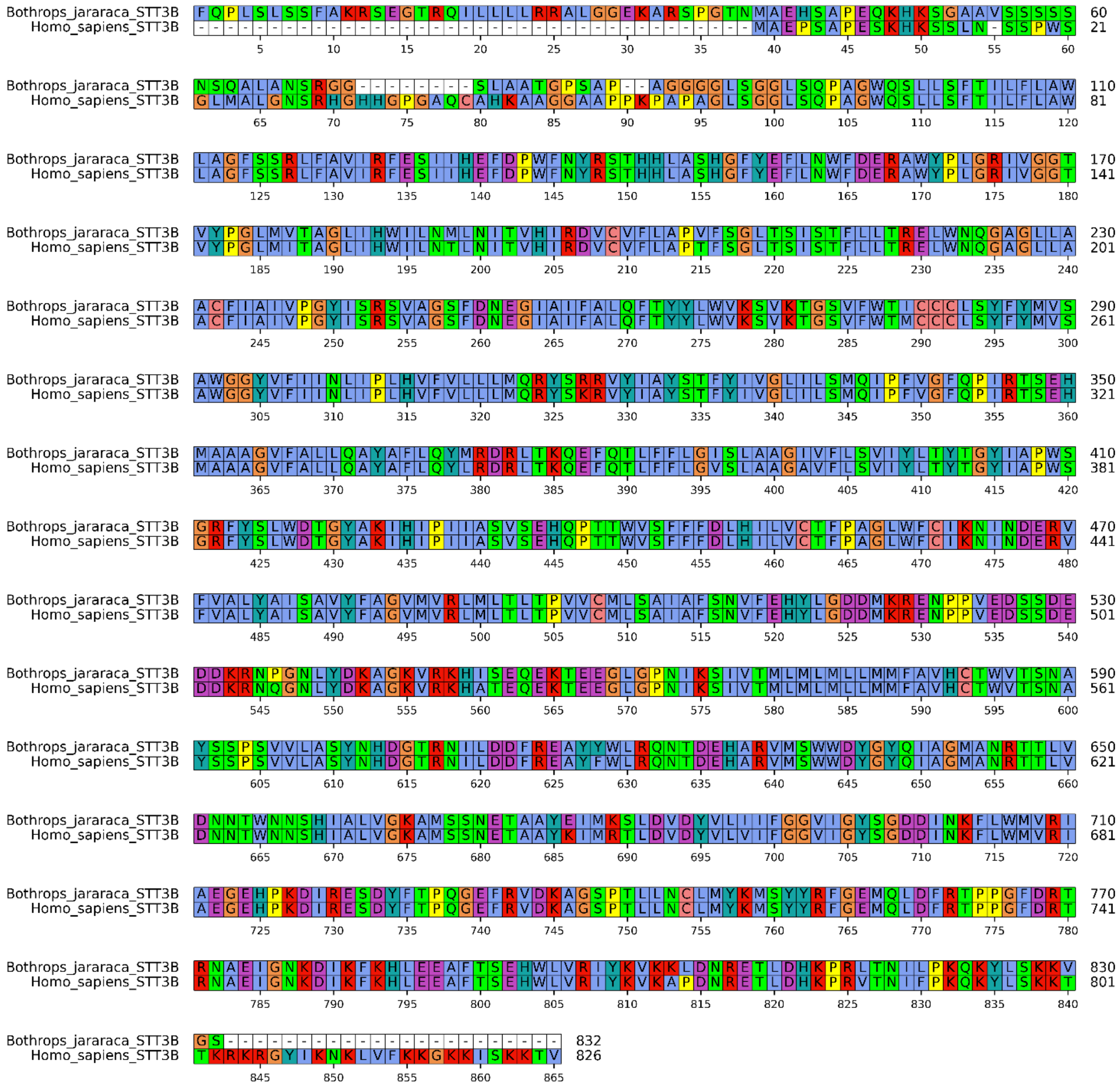

Figure S8. Sequence alignment between STT3B from *B. jararaca* and humans (UniProt/Swiss-Prot: Q8TCJ2).

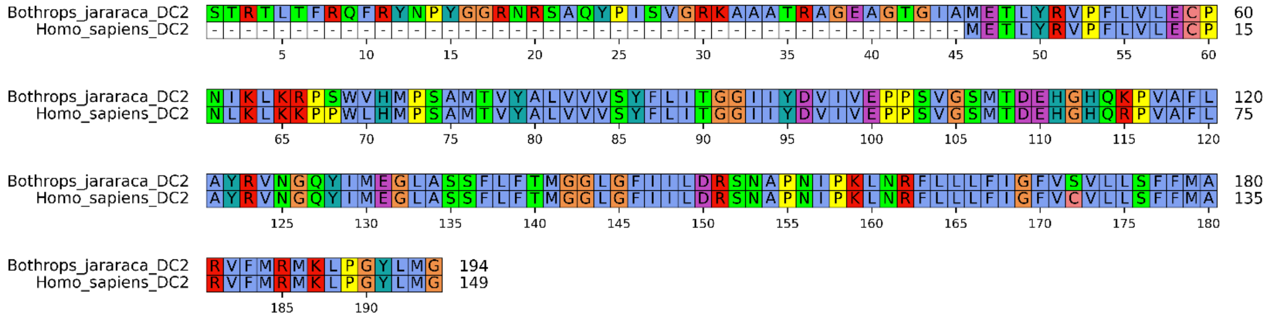

Figure S9. Sequence alignment between DC2 from *B. jararaca* and humans (UniProt/Swiss-Prot: Q9NRP0).

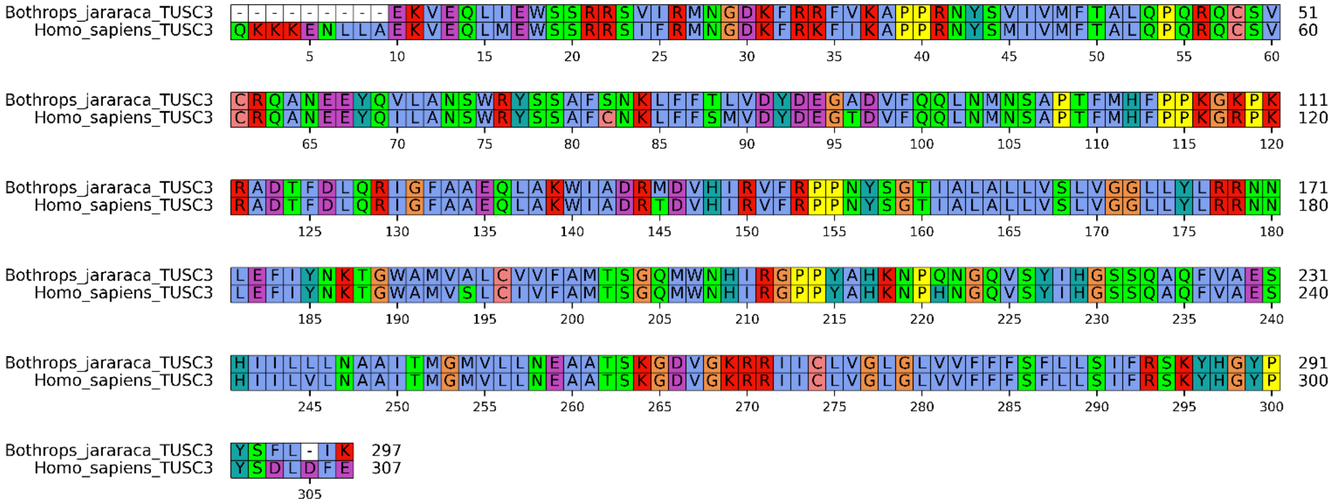

Figure S10. Sequence alignment between TUSC3 from *B. jararaca* and humans (UniProt/Swiss-Prot: Q13454).

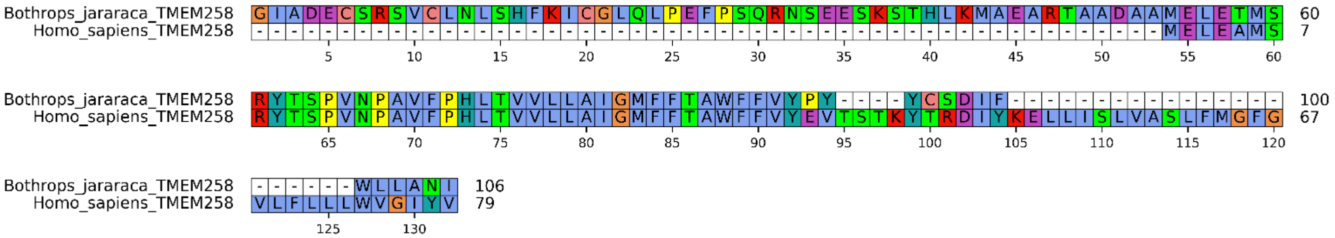

Figure S11. Sequence alignment between TMEM258 from *B. jararaca* and humans (UniProt/Swiss-Prot: P61165).

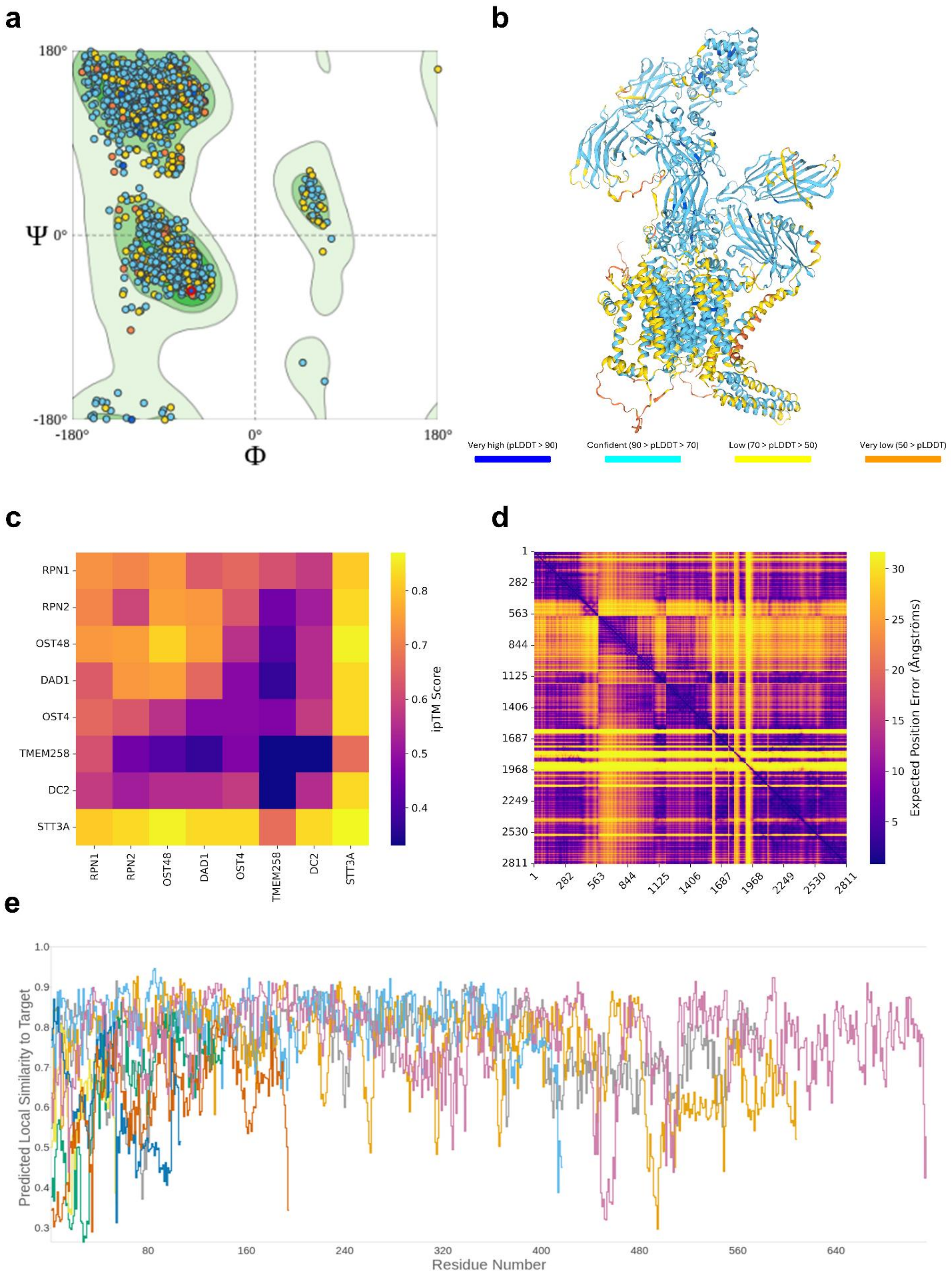

Figure S12. Quality Assessment of DC2-containing *B. jararaca* OST. **a** Ramachandran diagram. 97.96% of the residues (circles) were found in favored regions. **b** Model confidence. **c** iPTM score. **d** Expected Position Aligned Error (PAE). **e** QMEANDisCo local quality estimate. The model achieved a MolProbity score of 2.68 and a QMEANDisCo score of 0.75±0.05.

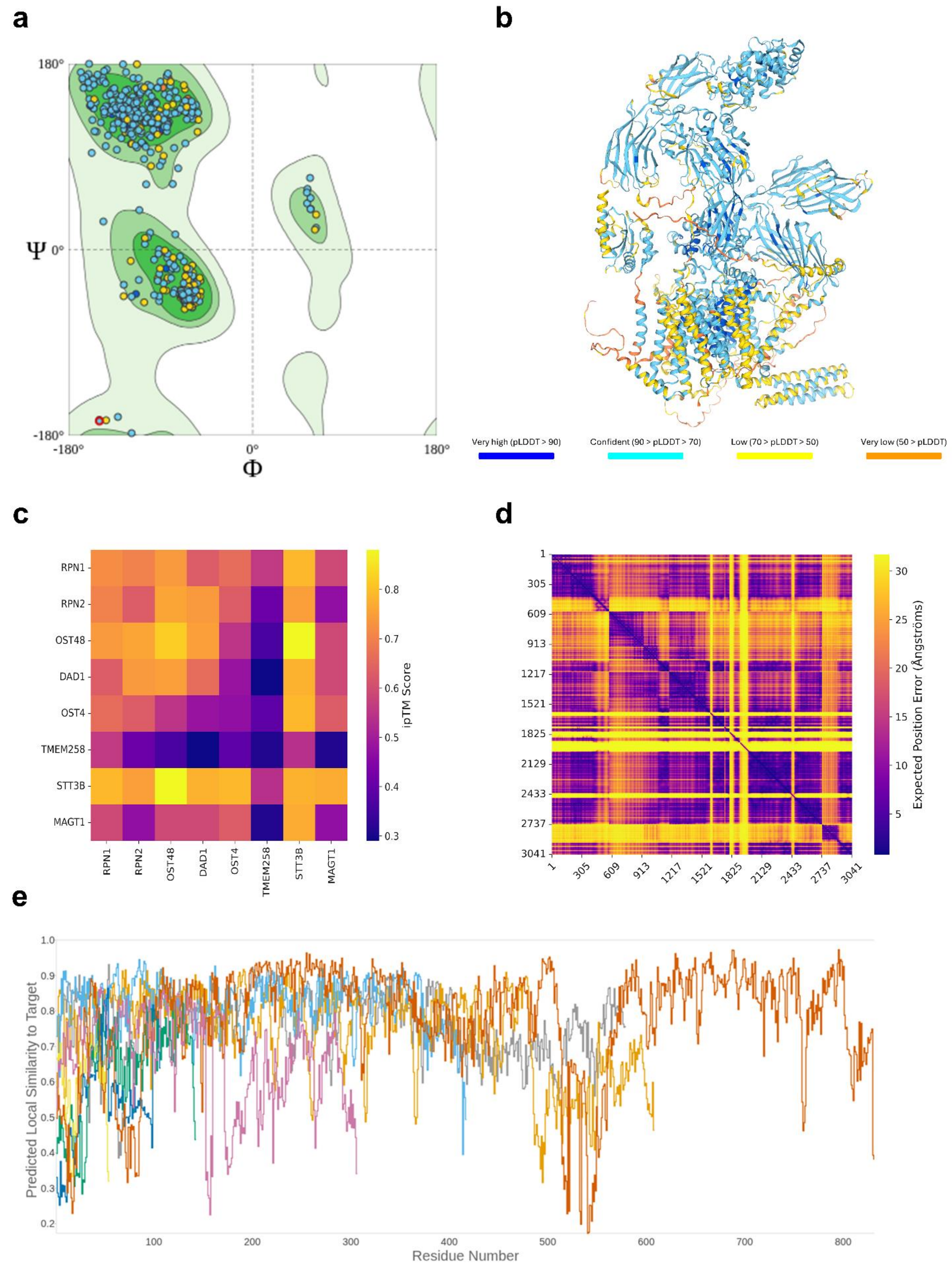

Figure S13. Quality Assessment of MAGT1-containing *B. jararaca* OST. **a** Ramachandran diagram. 97.82% of the residues (circles) were found in favored regions. **b** Model confidence. **c** iPTM score. **d** Expected Position Aligned Error (PAE). **e** QMEANDisCo local quality estimate. The model achieved a MolProbity score of 2.70 and a QMEANDisCo score of 0.75±0.05.
